# Effects of motor imagery-based brain-computer interface-controlled electrical stimulation on lower limb function in hemiplegic patients in the acute phase of stroke: a randomized controlled study

**DOI:** 10.3389/fneur.2024.1394424

**Published:** 2024-08-30

**Authors:** Xi Luo

**Affiliations:** ^1^North Sichuan Medical College, Nanchong, China; ^2^Pan Zhihua Integrated Traditional Chinese and Western Medicine Hospital, Panzhihua, China

**Keywords:** brain-computer interface, acute ischemic stroke, rehabilitative care, lower extremity motor dysfunction, BCI

## Abstract

**Background:**

Lower limb motor dysfunction is one of the most serious consequences of stroke; however, there is insufficient evidence for optimal rehabilitation strategies. Improving lower limb motor function through effective rehabilitation strategies is a top priority for stroke patients. Neuroplasticity is a key factor in the recovery of motor function. The extent to which neuroplasticity-based rehabilitation therapy using brain-computer interface (BCI) is effective in treating lower limb motor dysfunction in acute ischemic stroke patients has not been extensively investigated.

**Objective:**

This study aimed to assess the impact of BCI rehabilitation on lower limb motor dysfunction in individuals with acute ischemic stroke by evaluating motor function, walking ability, and daily living activities.

**Methods:**

This study was conducted in a randomized controlled trial, involving 64 patients with acute ischemic stroke who experienced lower limb motor dysfunction. All patients were divided into two groups, with 32 patients assigned to the control group was given conventional rehabilitation once a day for 70 min, 5 times a week for 2 weeks, and the experimental group (*n* = 32) was given BCI rehabilitation on top of the conventional rehabilitation for 1 h a day, 30 min of therapy in the morning and an additional 30 min in the afternoon, for a total of 20 sessions over a two-week period. The primary outcome was lower extremity motor function, which was assessed using the lower extremity portion of the Fugl-Meyer Rating Scale (FMA-LE), and the secondary endpoints were the Functional Ambulation Scale (FAC), and the Modified Barthel index (MBI).

**Results:**

After 20 sessions of treatment, both groups improved in motor function, walking function, and activities of daily living, and the improvements in FMA-LE scores (*p* < 0.001), FAC (*p* = 0.031), and MBI (*p* < 0.001) were more pronounced in the experimental group compared with the control group.

**Conclusion:**

Conventional rehabilitation therapy combined with BCI rehabilitation therapy can improve the lower limb motor function of hemiplegic patients with stroke, enhance the patient’s ability to perform activities of daily living, and promote the improvement of walking function, this is an effective rehabilitation policy to promote recovery from lower extremity motor function disorders.

## 1 Introduction

Walking function impairment is one of the most adverse effects of stroke. Approximately, 70–80% of patients in China are unable to live independently due to disability, and even in the chronic stage following stroke, 30% of patients are unable to walk, a key determinant of the chronic disability of stroke patients is motor dysfunction of the lower extremities (1–5). This dysfunction leads to reduced mobility and limits daily activities, increasing the risk of cardiovascular disease (6). These limitations not only reduce the quality of life of stroke patients with hemiplegia but also impose a huge financial burden on society, patients, and their families (7, 8). Moreover, lower limb motor dysfunction can have negative effects on a patient’s mental health by reducing self-confidence, as well as potentially leading to depression, pessimism, and other psychological issues (9). Therefore, rehabilitation of lower limb motor impairment is among the most urgent needs of stroke patients, and exploring more effective rehabilitation methods to improve the quality of patient survival is a long-term exploration goal in the field of stroke rehabilitation.

Routine rehabilitation promotes improvement of lower limb motor function to some extent, but there are certain shortcomings. Conventional exercises often focus on distal limb conditioning, neglecting the crucial role of central brain neuroplasticity. Consequently, even with intensive training, 15–30% of stroke patients may experience permanent disability (10, 11). Additionally, enhancing brain neuroplasticity can improve stroke patients’ motor function recovery. Neuroplasticity is the neurophysiological basis for the recovery of bodily function after CNS injury, and it is the most critical driving factor for the recovery of motor function after stroke, it is considered to be the mechanism for the recovery of functional movement in patients with ischemic stroke, and it plays an important role in rehabilitation (12–15).

The Brain-Computer Interface (BCI) is a therapeutic approach based on the principle of neuroplasticity to promote motor function rehabilitation in stroke patients (10). Brain-computer interface rehabilitation therapy combines motor imagery therapy with physical therapy. It works by capturing the brain’s intention of motor imagery, using functional electrical stimulation to stimulate the affected limbs, and providing feedback on the results of motor imagery through proprioception. This creates a closed-loop central-peripheral-central rehabilitation model that promotes neuroplasticity and the restoration of motor function (16).

Rehabilitation of lower limb motor function is one of the most urgent needs of stroke patients. Still, the use of electrical stimulation therapy controlled by a brain-computer interface based on motor imagery for the rehabilitation of lower limb function is relatively rare, and the effect on the rehabilitation of patients in the acute phase of stroke is still being explored. It has been proposed that neuroplasticity is most active in the acute phase, so early post-stroke rehabilitation should begin with implementing effective interventions (17). Therefore, this study aims to investigate the effects of electrical stimulation therapy controlled by a brain-computer interface based on motor imagery on rehabilitating lower limb motor function in hemiplegic patients during the acute phase of ischemic stroke.

The study also aims to evaluate the impact of BCI on the ability to perform daily activities and walk in hemiplegic stroke patients.

## 2 Methods

### 2.1 Participants and study design

This study was a single-center, prospective, randomized controlled clinical trial conducted from December 2021 to November 2022. It was approved by the Ethics Committee of the Affiliated Hospital of Chuanbei Medical College (IRB number: 2022ER172-1) and funded by the Nanchong Social Science Research Program (Grant NC23C141).

Both the experimental and control groups received routine medications during the treatment period. Control group: conventional rehabilitation (conventional rehabilitation training + conventional rehabilitation care).

Conventional rehabilitation training: includes physical therapy (40 min) and acupuncture treatment (30 min). Physical therapy is mainly Bobath manipulation training techniques, gait correction training, etc. Acupuncture treatment was administered by a professional acupuncture therapist. Conventional rehabilitation care: Involves proper limb position placement (18, 19), limb massage (20), and psychological care (21).

Participants in the experimental group received BCI rehabilitation in addition to conventional rehabilitation for 1 h per day, 30 min in the morning, and 30 min in the afternoon for 2 weeks, for a total of 20 sessions.

### 2.2 Inclusion and exclusion criteria

**Figure 1:**
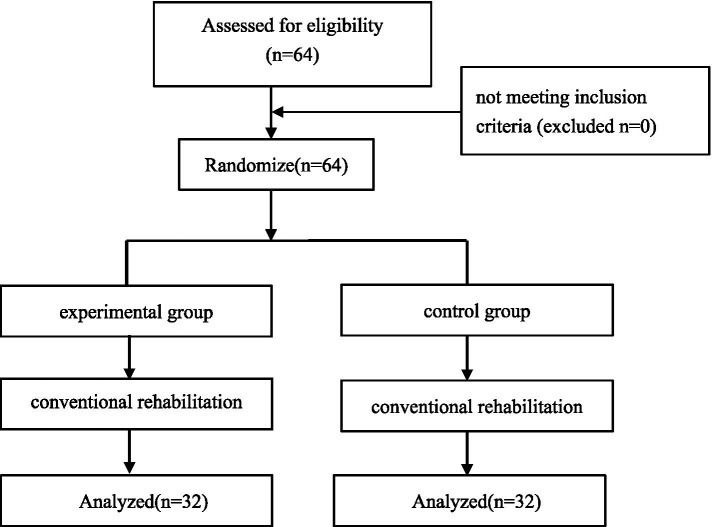
Consolidated standards of reporting trials flow diagram.

Subjects were recruited to the Department of Neurology. All participants met the diagnostic criteria of the Chinese Guidelines for Diagnosis and Treatment of Acute Ischemic Stroke 2018 (22), and the diagnosis was confirmed by cranial CT/MRI imaging. Inclusion criteria were patients with a disease duration of 2 weeks or less, first diagnosed with acute cerebral infarction, unilateral hemiparesis, and motor dysfunction of the affected lower limb; Brunnstrom’s lower limb staging stage II-IV; seated balance ≥ grade 1; aged 18–70 years old; stable vital signs, stabilized condition, clear mentation, and no verbal communication disorders; a score of ≥ 21 on the Brief Mental State Examination (MMSE); National Institutes of Health Stroke Scale (NIHSS) score ≤ 15 points; signed informed consent for treatment. Excluded were those with severe cardiac, hepatic, renal diseases and malignant tumors in combination; those with a previous history of epileptic seizures; those with other skeletal-muscular or neurological diseases affecting the recovery of motor function; the presence of electrical, magnetic, or other metal implants; cranial defects; visual field defects; and venous thrombosis in the lower limbs. The participant flow chart is shown in Figure 1.

### 2.3 Sample size calculations

The sample size was calculated using the lower extremity portion of the Fugl-Meyer Rating Scale (FMA-LE), the primary outcome indicator, using G*power 3.1.9.7 software. According to a previous study (23) FMA-LE in the experimental and control groups after the intervention were: 22.50 ± 3.27 and 20.00 ± 2.41 respectively, the study set Effect size. f to 0.87, Type I error α = 0.05, and Type II error *β* = 0.1, and the data were entered into this software to give *n* = 58, after taking into account the 10% dropout rate, the number of cases required for each group was obtained to be 32, with a total sample size of 64. Patients who met the inclusion criteria were made aware of the purpose and procedures of the program and signed an informed consent form.

Patients who agreed to participate were randomly assigned to the control and experimental groups. Block group randomization was used to group the selected subjects. Before grouping, randomized envelopes with sequential numbering were developed. After signing the informed consent for cases that met the inclusion and exclusion criteria for this study, the envelopes were opened by non-participants. The enrollment of patients was determined according to the allocation scheme in the envelopes, and the appropriate interventions were selected.

### 2.4 Outcome indicator

The clinical assessment was conducted by an independent assessor from the Department of Neurology, who completed a general information questionnaire before and after treatment, which included gender, side of hemiparesis, age, height, weight, duration of illness, and NIHSS score (24), the NIHSS is a reliable, valid, and responsive measure of stroke severity that helps clinicians provide patients with accurate information and set realistic treatment goals (25). Fugl-Meyer assessment of Lower Extremity (FMA-LE) (26, 27), Functional Ambulation Category Scale (FAC) (28), Modified Barthel index (MBI) (29). The primary outcome indicator was the Fugl-Meyer assessment of Lower Extremity and the secondary outcome indicators were the Functional Ambulation Category Scale and the Modified Barthel index.

### 2.5 BCI protocol

**Figure 2:**
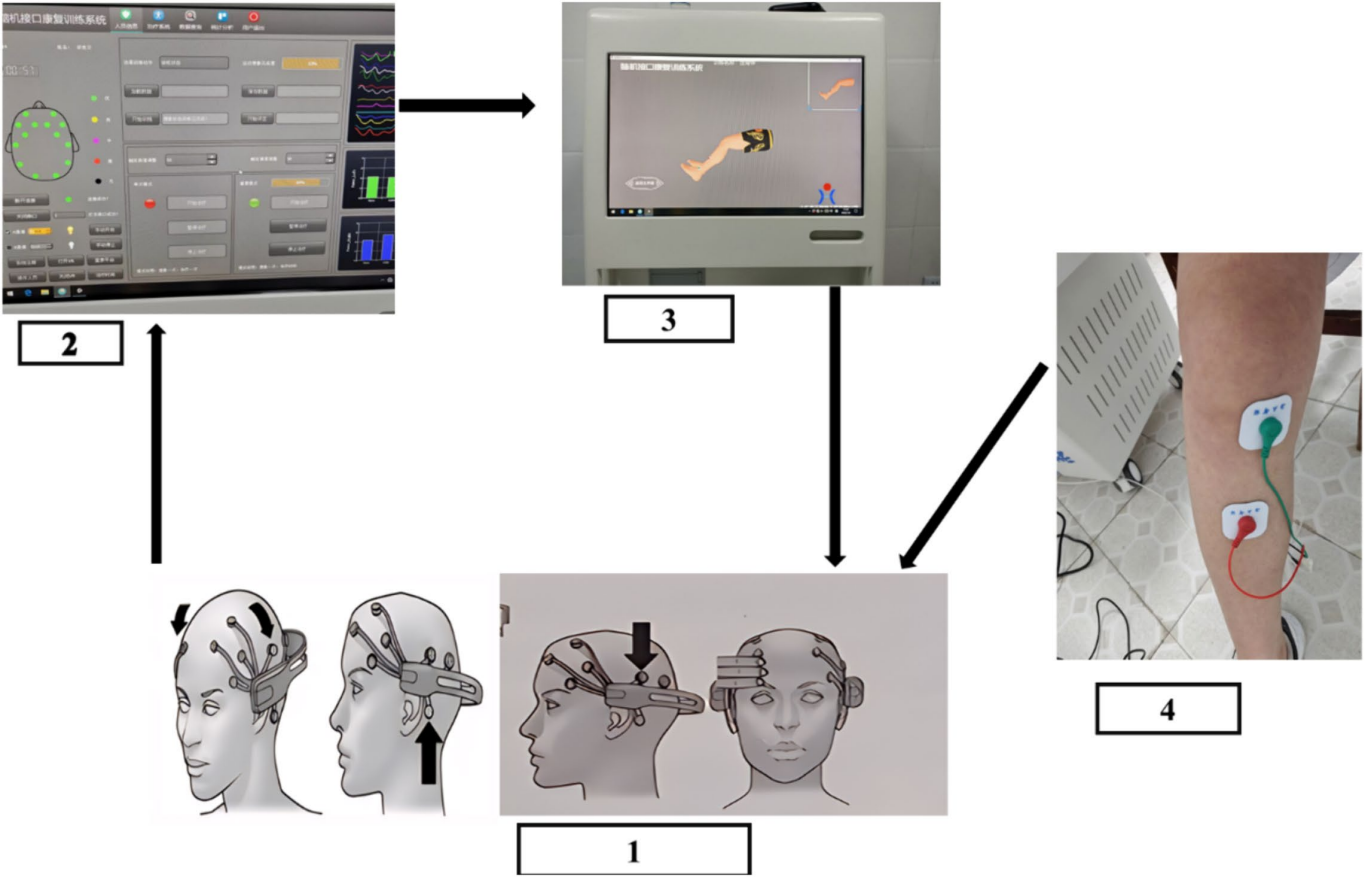
Flow chart of BCI training: (1) Wear an EEG controller to collect EEG signals. (2) Classifying and decoding motion intentions (3) visual aid. (4) Functional electrical stimulation.

The design of the BCI-controlled functional electrical stimulator is shown in Figure 2.

BCI rehabilitation: the LSR-AII brain-computer interface rehabilitation training system (Shandong Haitian Intelligence) was used for foot dorsiflexion training, knee extension training, foot inversion training, and externally rotated calf training.

① Plug in the plug, turn on the power supply and the host switch, insert the USB Bluetooth connection, open the rehabilitation system, enter the patient’s information, and then have the patient sit on the chair or wheelchair facing the treatment interface.

② Ensure that the EEG controller has sufficient power by checking the green indicator light. Dampen the sponge electrode head with a coupling agent to ensure it is moist enough, and then install the electrode head onto the EEG cap one by one in empty spaces.

③ Wearing EEG controller: turn on the EEG controller switch, click on the EEG connection, and slide the installed EEG cap gently and slowly from the patient’s head, the rubber electrode head is located in the bilateral and posterior mastoid process, the forehead electrode is located in the hairline, about three transverse fingers on the eyebrow, adjusting the electrode position, to ensure that on the rehabilitation system, the connection of each EEG signal is in the green state, indicating that the contact effect is superior.

④ According to the treatment plan, electrode sheets are pasted on the corresponding muscle groups, and the current size is adjusted to be tolerated by the patient, to avoid the current being too large and causing harm to the patient.

⑤ MI training through voice and VR screen, computerized assessment of motor imagery with at least 50% accuracy.

⑥ BCI rehabilitation can only be performed after motor imagery reaches the standard.

⑦ The threshold of motor imagery in BCI rehabilitation is 30%, and reaching the threshold triggers functional electrical stimulation to stimulate the muscles to produce the corresponding movements, on the contrary, if the motor imagery does not reach the threshold, functional electrical stimulation cannot be initiated, and voice prompts will appear to improve the patient’s attention and prepare for the next motor imagery.

The movement imagery for this study was foot dorsiflexion, foot inversion, knee extension, and calf external rotation, and the primary muscle groups stimulated were: tibialis anterior, gastrocnemius, quadriceps femoris, and biceps femoris.

Foot dorsiflexion training: After the VR data acquisition is completed, the rehabilitation system displays the patient’s movement imagination completion degree, which should reach at least 30. Then, following the animated prompts, the electrode sheet is applied to the corresponding muscle groups. The serial port is opened, and the current size is adjusted, starting from 0 and increasing gradually, while ensuring the patient does not experience discomfort. The treatment mode is set to repeat, with each treatment lasting for 60 s. The minimum trigger value for each patient is 50, and the treatment time for foot dorsiflexion training is 15 min, after which the system automatically stops. Knee extension training: according to the VR animation prompts the electrode sheet will be attached to the corresponding muscle groups, and the current size, the patient according to the VR screen action prompts, movement imagination, imagination success once, 60 s of rehabilitation, knee extension training treatment time is 15 min, 15 min rehabilitation is completed automatically stop. Foot inversion training: according to the VR animation prompts the electrode sheet is affixed to the corresponding muscle group, and the current size, the patient according to the VR screen action prompts, movement imagination, imagination success once, 60 s of rehabilitation therapy, foot inversion training treatment time is 15 min, 15 min of rehabilitation is completed automatically stop. External rotation calf training: according to the VR animation prompts the electrode sheet will be attached to the corresponding muscle group, the current size, and the patient according to the VR screen action prompts, exercise imagination, imagination success once, 60 s of rehabilitation therapy, external rotation calf training treatment time is 15 min, 15 min after the completion of rehabilitation is automatically stopped.

### 2.6 Statistical analysis

SPSS25.0 software was used for statistical analysis, and the measurement data used in this study were expressed as mean plus minus standard deviation (x ® ± s), normality test and variance chi-square test, *t*-test if it meets the requirements, and vice versa rank-sum test; the count data were described by frequency and constitutive ratio using *x*^2^ test; and the hierarchical data were utilized by the two-sample comparisons of the Wilcoxon rank-sum test. Statistical tests were performed using two-sided tests, and differences were considered statistically significant at *p* < 0.05.

## 3 Results

A total of 64 patients met the inclusion–exclusion criteria and were randomized. Table 1 shows general data of patients in both groups, and there were no statistically significant differences between the two groups in terms of basic demographic and clinical characteristics (including gender, age, disease duration, height, weight, hemiplegic side, and NIHSS score) (*p* > 0.05). In addition, only one patient responded with fatigue, which disappeared after rest, and no other adverse events occurred.

**Table 1:** Comparison of the general data of the two groups of patients.

	Experimental group	Control group	*x*^2^/*t/z* value	*p* value
	(*n* = 32)	(*n* = 32)		
Sex (M/F)	23/9	19/13	1.108^b^	0.292
Paretic side (L/R)	18/14	15/17	0.563^b^	0.453
Age (years)			1.730^b^	0.421
≤40	2 (6.3%)	1 (3.1%)		
41–60	19 (59.4%)	15 (46.9%)		
>60	11 (34.4%)	16 (50%)		
Height	162.59 ± 5.19	160.13 ± 6.04	1.754^a^	0.084
Weight	65.03 ± 7.49	61.53 ± 6.72	1.967^a^	0.054
Length of stay	13.72 ± 1.51	15.03 ± 3.40	1.904^a^	0.064
NIHSS	4.25 ± 1.32	4.53 ± 1.430	0.816^a^	0.418
FMA-LE	7.94 ± 1.24	8.06 ± 1.24	0.402^a^	0.689

^a^The independent samples *t*-test. ^b^The *x*^2^ test.

### 3.1 Comparison of Fugl-Meyer assessment of lower extremity scores between the two groups of patients

**Figure 3:**
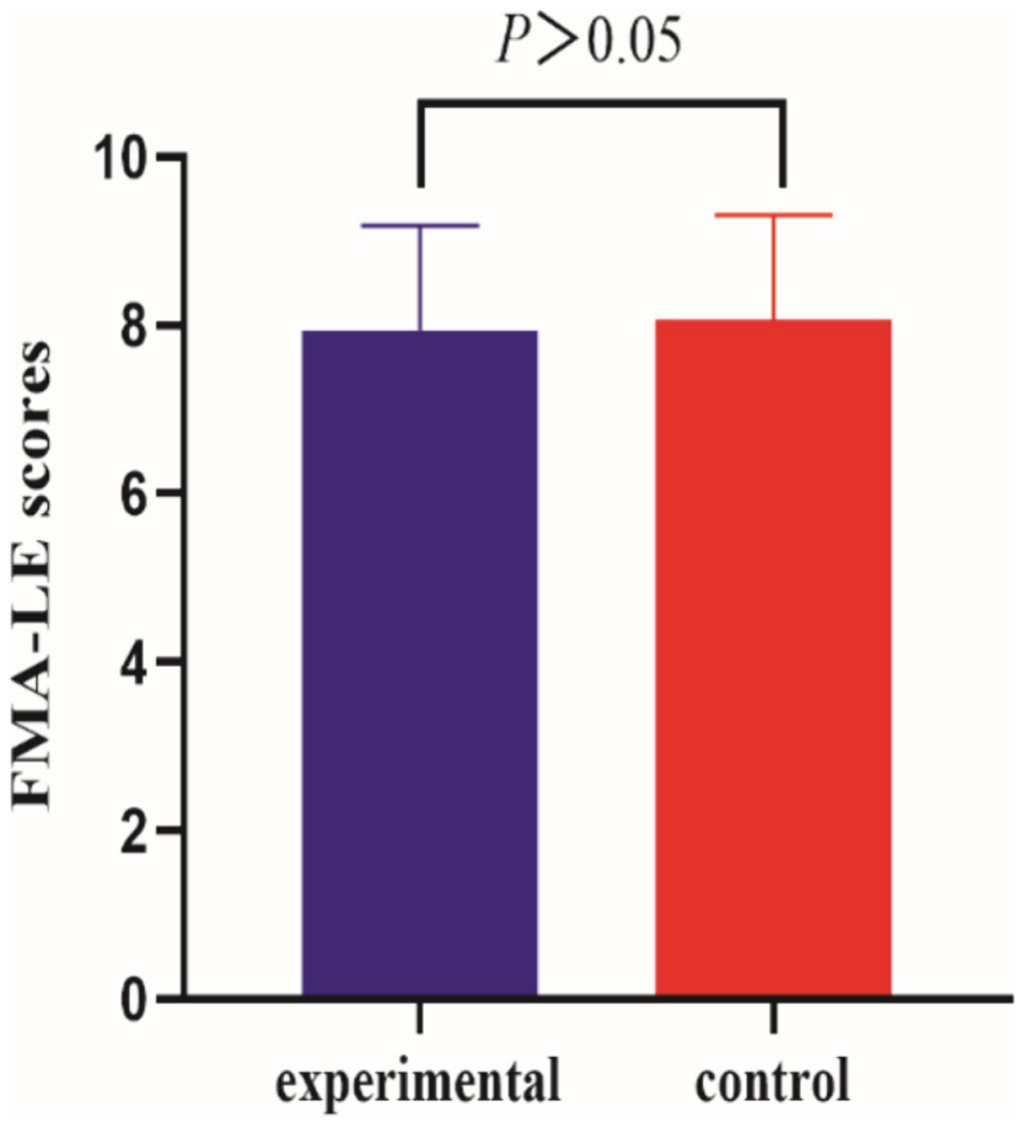
Comparison of FMA-LE scores between the two groups of patients pre-intervention.

**Figure 4:**
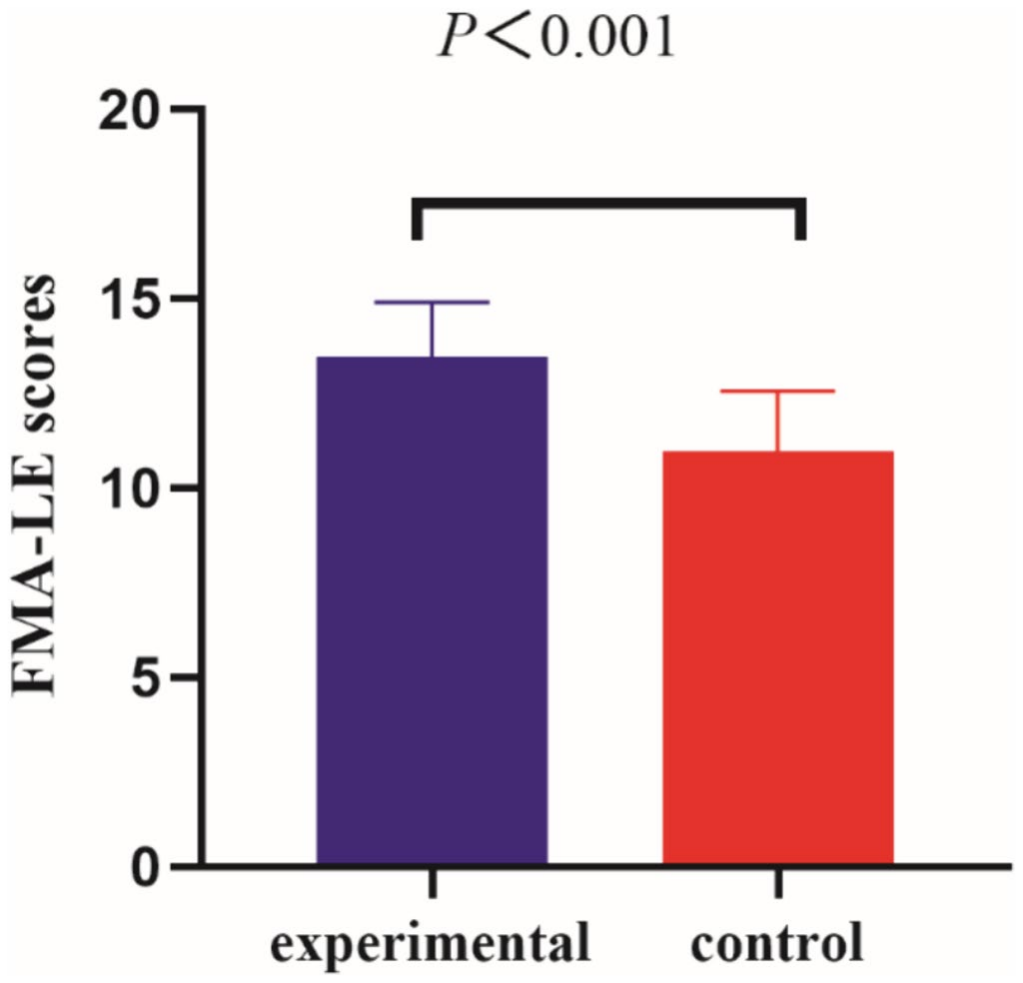
Comparison of FMA-LE scores between the two groups of patients post-intervention.

Before the intervention, the FMA-LE scores of the two groups were compared, and the difference was not statistically significant (*p* > 0.05); after the intervention, the difference between the FMA-LE scores of the experimental group compared to the control group was statistically significant (*p* < 0.001) (see Table 2 and Figures 3, 4).

**Table 2:** Comparison of Fugl-Meyer score of lower limb motor function scores before and after intervention in the two groups (^−^*x* ± *s*).

	Numbers	Experimental	Control	*t*	*p*
Pre-intervention group	32	7.94 ± 1.24	8.06 ± 1.24	0.402	0.689
Post-intervention	32	13.47 ± 1.44	10.97 ± 1.60	6.586	*p* < 0.001

Using the Bonferroni correction method, the results demonstrate *F* = 43.38; *p* < 0.001, suggesting a significant statistical distinction between the experimental and control groups.

### 3.2 Comparison of functional ambulation category scale between two groups of patients

Before the intervention, the FAC grading index of the two groups of patients was compared, and the difference was not statistically significant (*p* > 0.05); after the intervention, there was a statistically significant difference in the FAC grading index in the experimental group compared to the control group (*p* = 0.031); the FAC grading index was higher in the experimental group after the intervention than before the intervention, and the difference was statistically significant (*p* < 0.001); the difference in the FAC grading index after the intervention in the control group compared to the pre-intervention period was statistically significant (*p* = 0.001). As shown in Tables 3, 4.

**Table 3:** Comparison of FAC scale before and after intervention in both groups (number).

Time	Group	FAC class						*z* value	*p* value
		0	1	2	3	4	5		
Pre-intervention								0.872	0.383
Experimental group		19	9	4	0	0	0		
Control group		22	8	2	0	0	0		
Post-intervention								2.151	0.031
Experimental group		7	14	5	4	1	0		
Control group		15	13	2	2	0	0		

Comparison between pre-intervention and post-intervention in the experimental group, *z* = 4.413, *p* = 0.000, and between pre-intervention and post-intervention in the control group, *z* = 3.317, *p* = 0.001.

**Table 4:** Logistic regression analysis of length of hospitalization, age, and post-intervention FAC.

	OR	*p*	95% CI
Age	1.05	0.120	−0.013 – 0.109
Length of stay	0.98	0.785	−0.203 – 0.153

### 3.3 Comparison of Modified Barthel index scale scores between the two groups of patients

**Figure 5:**
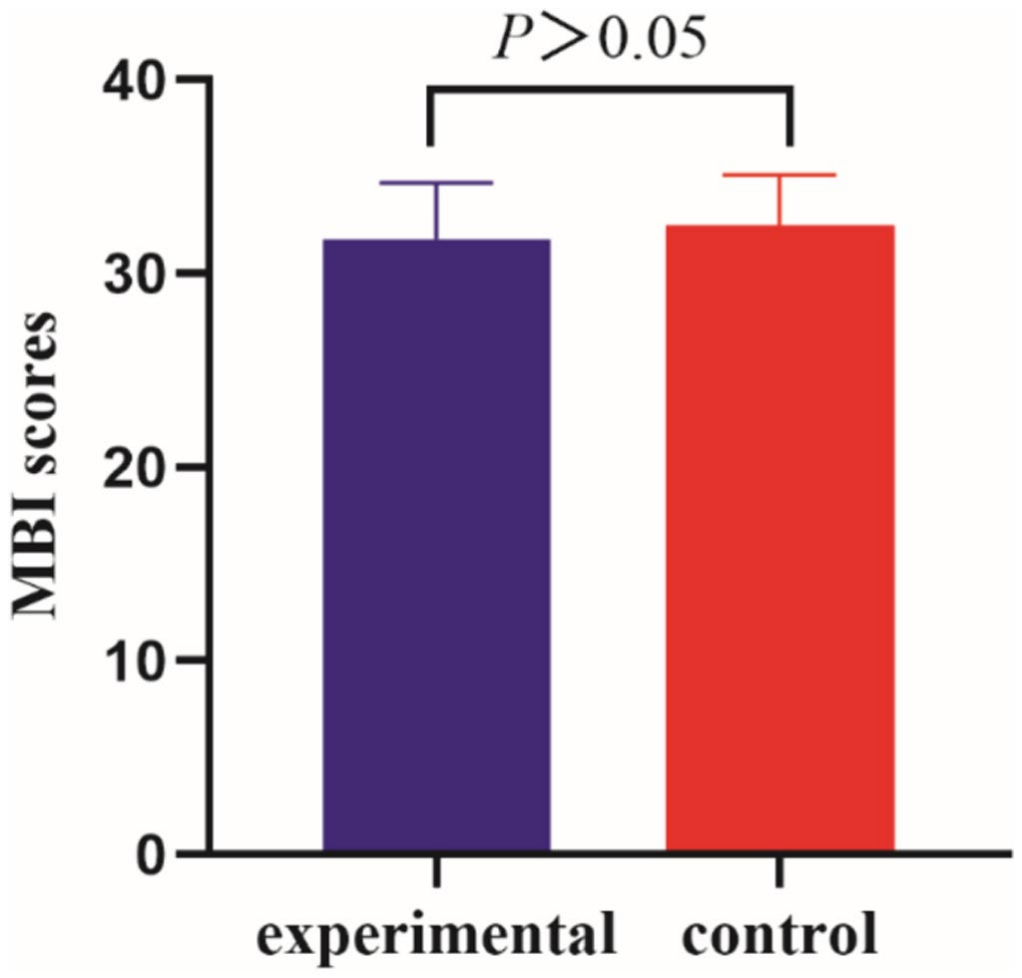
Comparison of MBI between the two groups of patients pre-intervention.

**Figure 6:**
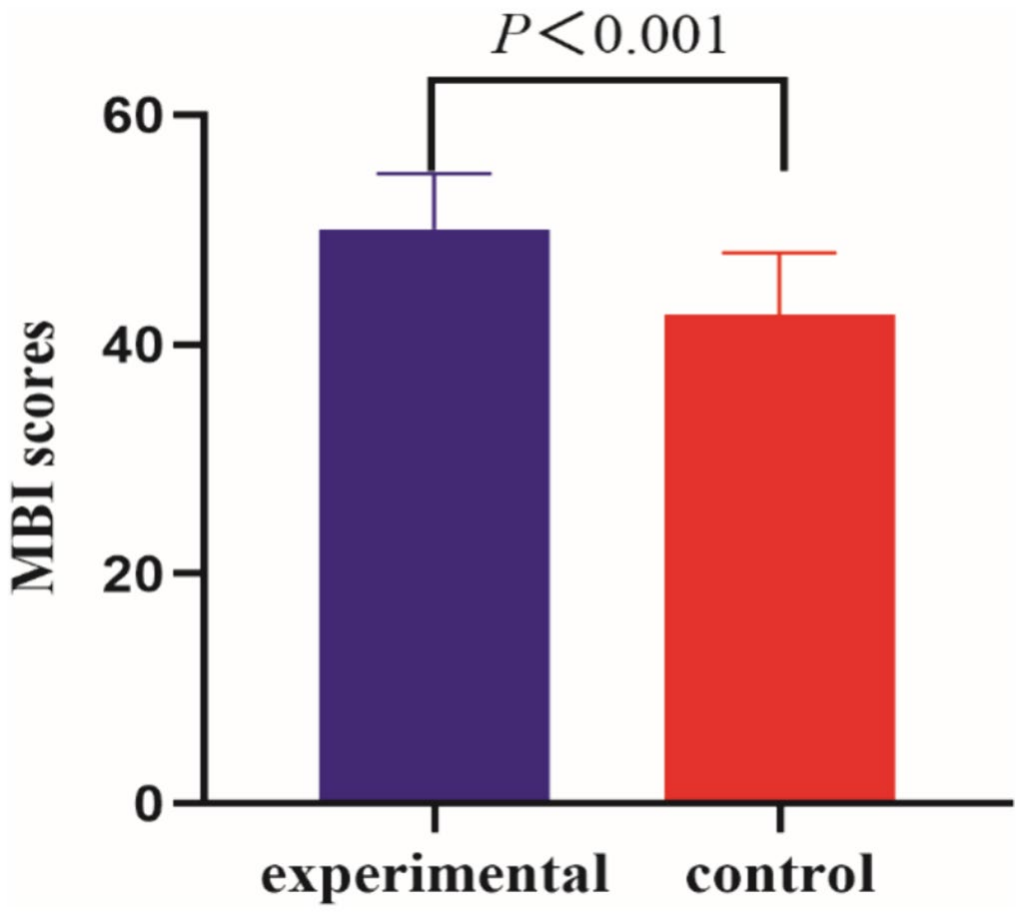
Comparison of MBI between the two groups of patients post-intervention.

Before the intervention, the MBI scores of the two groups of patients were compared, and the difference was not statistically significant (*p* > 0.05); after the intervention, there was a statistically significant difference in the MBI scores of the experimental group compared to the control group (*p* < 0.001) (Tables 5, 6 and Figures 5, 6).

**Table 5:** Comparison of pre-intervention and post-intervention Barthel scores between the test and control groups [score, M (P25, P75)].

Group	Numbers	Pre-intervention	Post-intervention	*z* value	*p* value
Experimental group	32	31 (30, 33)	50 (47, 52)	4.955	0.000 ^b^
Control group	32	32 (31, 34)	40 (40, 43)	4.973	0.000 ^b^
*z* value		1.490	5.440		
*p* value		0.136	0.000 ^a^		

^a^Indicates a statistically significant difference of *p* < 0.05 when compared with the control group after the intervention.

^b^Indicates a statistically significant difference compared to the same group before treatment, *p* < 0.05.

**Table 6:** MBI covariance analysis

Group	Numbers	Pre-intervention	Post-intervention	*F*	*p*
Experimental group	32	31.781 ± 2.915	50.535 ± 0.554	115.904	0.000
Control group	32	32.500 ± 2.577	42.059 ± 0.554		

## 4 Discussion

The results of this study suggest that the combination of motor imagery-based brain-computer interface (BCI) controlled electrical stimulation and conventional rehabilitation has a beneficial effect on the lower limb motor function, walking ability, and activities of daily living in patients recovering from acute ischemic stroke (17). A Meta-analysis study showed that intervention during the acute phase better-improved motor function and activities of daily living in stroke patients (30). These study findings further validate the potential advantages of integrating motor imagery-based BCI-controlled electrical stimulation with standard rehabilitation in enhancing lower limb motor function and daily activities in stroke patients. This holistic intervention approach may expedite the patients’ recovery journey and enhance their overall quality of life.

### 4.1 The effect of brain-computer interface combined with conventional rehabilitation on lower limb functioning

The Chinese Stroke Rehabilitation Guidelines (5) recommend that rehabilitation should be done as early as possible after the patient’s condition is stabilized to provide early rehabilitation and improve muscle strength and function of the paralyzed limbs. Motor imagery therapy can be used at any stage of recovery for hemiplegic stroke patients, giving full play to the patient’s subjective initiative, and more in line with the transmission from the brain to the limbs, from top to bottom, to activate, improve, and strengthen the stroke-damaged neural network, to improve the patient’s hemiplegic side of the limb function (30).

In this study, routine rehabilitation care and rehabilitation training were used in the control group, and the patient’s lower limb function improved after the intervention. Wang (31) used early rehabilitation care for stroke patients, and the results showed that the FMA score of the experimental group was higher than that of the control group, and rehabilitation care could effectively promote the improvement of patients’ motor function. Another study showed that the value of the clinical use of early rehabilitation care to improve limb function in stroke patients is worth recognizing (32). The reason is that early rehabilitation care through the placement of good limb position, and limb massage, can help patients prevent muscle atrophy, and joint deformation, improve limb spasms, and promote the recovery of patient’s motor function, psychological care, and health education can help patients to correctly recognize the disease, to reduce the fear, and to enhance the patient’s confidence in recovery. Therefore, early rehabilitation care has significant nursing value for improving motor function in stroke patients.

A study proposed a minimum clinically important difference (MCID) of 6 points for FMA-LE (33). In this study, 43.8% of all patients undergoing BCI rehabilitation achieved a minimum clinically important difference in FMA-LE scores, with an average score of 6.64 for FMA-LE. Compared to the control group post-intervention, the experimental group underwent brain-computer interface controlled electrical stimulation rehabilitation training, resulting in better improvement in lower limb motor function in stroke survivors with hemiplegia. The experimental results are consistent with similar previous research findings. Yuan et al. (34) applied brain-computer interface-controlled stepping training to the rehabilitation of lower limb motor function in stroke patients for 2 weeks, 6 times per week, and the FMA-LE scores of the test group improved significantly compared to the control group. Chung et al. (35) applied BCI-FES to the rehabilitation of lower limb motor function for 30 min per day for 5 days and showed that BCI-FES improved gait function better in patients. The reasons are as follows (10, 36–38): the brain-computer interface-controlled electrical stimulation rehabilitation therapy based on motor imagery combines motor imagery therapy with physical therapy, forming a closed-loop treatment mode of central-peripheral-central. ① When the patient imagines the correct movement, the system detects the appropriate EEG signals and sends out electrical stimulation to carry out actual motor therapy on the paralyzed limb. The movement of the paralyzed limb provides a large number of proprioceptive input impulses to the central nervous system, increasing the excitability of the nerve cells in the damaged and surrounding areas. This promotes the repair of damaged cells and the compensatory function of the terminally damaged cells. The closed-loop rehabilitation mode synchronizes the activation of the cerebral motor cortex and the peripheral effectors, inducing Hebbian plasticity and promoting the restoration of motor function. ② Motor imagery is one of the keys to BCI treatment, and patients need correct motor imagery to enter rehabilitation. A large amount of correct motor imagery can activate potential neuronal pathways and dormant synapses, improve cerebral blood flow, activate the corresponding functional brain areas, strengthen cerebral cortical connections, and promote functional reorganization of the brain, which will lead to the improvement of lower limb motor function. ③ The patient repeats BCI rehabilitation to activate the body’s natural efferent and afferent channels, and the closed-loop rehabilitation strengthens the sensory and motor circuits, promotes motor learning and neural plasticity, and thus facilitates the recovery of motor function. ④ Compared with other passive training, BCI rehabilitation requires the active participation of the patient to carry out the treatment, and the active participation of the patient mobilizes the patient’s motivation for rehabilitation, improves the cooperation of the patient’s rehabilitation, and therefore enhances the therapeutic effect. ⑤ BCI-FES combines motor imagery with functional electrical stimulation, with both central and peripheral interventions, forming a top-down closed-loop rehabilitation approach, where the center promotes peripheral muscle activity, and the periphery feeds feedback back to the center to promote remodeling of the brain’s function. Brain-computer interface rehabilitation stimulates both the central nervous system and the muscles, avoiding muscular atrophy and promoting the recovery of muscle strength. Therefore, the combination of BCI-FES rehabilitation based on motor imagery based on conventional rehabilitation can better promote the rehabilitation of lower limb motor function in stroke patients with hemiplegia.

### 4.2 Effect of brain-computer interface combined with conventional rehabilitation on walking function

Walking is the basis of human activity and has an important impact on patients’ self-care, social activities, and quality of life, as well as return to their families to reintegrate into society and participate in social work, so the improvement of walking function is also an important outcome of the recovery of lower limb motor function.

In this study, conventional rehabilitation care and rehabilitation training were used in the control group, and the results showed that the walking function of the patients improved after the intervention. Conventional rehabilitation care can prevent patients’ muscle atrophy, relieve spasms, promote blood circulation, enhance the effect of rehabilitation intervention, and improve patients’ walking function through the placement of good limb position, massage, and other measures. A study showed that rehabilitative care can enhance the rehabilitation of patients’ walking function (39). In addition, the results of a Meta-analysis showed that early rehabilitative care can improve walking ability in stroke patients (40). Early rehabilitation care is important for the recovery of motor function in stroke patients and should be emphasized.

Compared with the post-intervention control group, the experimental group showed a more significant improvement in walking function, so the results of this study suggest that conventional rehabilitation combined with motor imagery brain-computer interface electrical stimulation rehabilitation can promote the improvement of walking function in hemiplegic patients with stroke. And no MCID has been reported on gait (41). A similar study used motor imagery therapy combined with electromyographic biofeedback for the rehabilitation of hemiplegic patients with stroke, and at the end of the intervention, the patients’ FAC scale ratings were better than those of the control group (42). The reason for this is that electrical stimulation therapy, controlled by a brain-computer interface based on motor imagery, improves walking function by stimulating the muscles, causing them to contract to move, improving muscle strength, and improving the function of the paralyzed limb. Stroke rehabilitation guidelines also suggest that acute stroke patients can improve muscle strength and paralyzed limb function with electrical stimulation and early rehabilitation (5). Other studies have also shown that BCI can promote improvements in patients’ walking ability (43). The above studies are consistent with the results of the present study; therefore, conventional rehabilitation combined with electrical stimulation rehabilitation with a brain-computer interface for motor imagery can promote the improvement of walking function in stroke patients.

### 4.3 Effects of brain-computer interface combined with conventional rehabilitation on activities of daily living ability

Stroke patients with lower limb motor dysfunction often have a decline in self-care ability, patients can not walk independently, need the help of others to toilet or go out, unable to participate in social activities on their own, in the long run, the patient is prone to anxiety, depression, loneliness, and other emotions, which increases the burden on the caregiver, the family, and the community.

The control group used conventional rehabilitation care and rehabilitation training, and the results showed that the patient’s activities of daily living improved after the intervention. The implementation of conventional rehabilitation care for patients, through psychological care to alleviate the patient’s adverse emotions, enhance the patient’s confidence in recovery, the placement of good limb position, and limb massage, help to improve motor function, thereby improving the ability to patients to carry out activities of daily living. Studies by Hangjian Qiu (40), Cumming (20), Im HW (44) have shown that early rehabilitation can improve the ability of stroke patients to perform activities of daily living. Therefore, early rehabilitative care plays an important role in the ability of stroke patients to perform activities of daily living.

The ability to perform activities of daily living was significantly improved in the experimental group after the intervention compared to the control group. The results showed that the addition of brain-computer interface (BCI) rehabilitation therapy was superior to conventional rehabilitation therapy and could achieve better rehabilitation effects, and the BCI rehabilitation therapy improved the muscle strength of the paralyzed limbs and facilitated the recovery of the motor function of the lower limbs, which in turn enhanced the daily life activities of the patients. It has been demonstrated that the minimum clinically important difference for MBI is >5.34 points (45). In this study, the minimum clinically important difference for MBI was also >5.34 points. Therefore, this study concluded that brain-computer interface rehabilitation therapy has an improving effect on patients’ ability to perform activities of daily living.

### 4.4 Study limitations

This study has certain limitations. Firstly, the population included in this study was patients in the acute stage cannot be extrapolated to other poststroke phases, and no long-term follow-up, Secondly, quantitative measurements such as fMRI and TMS were not integrated into this study to further validate the rehabilitation effects. Additionally, although the results of the present study were meaningful, the control group did not receive sham stimulation and failed to exclude certain confounding factors; therefore, a better way to validate this could be implemented in the future by implementing a sham stimulation intervention in the control group.

## Data availability statement

The original contributions presented in the study are included in the article/supplementary material, further inquiries can be directed to the corresponding author.

## Ethics statement

The studies involving humans were approved by the Ethics Committee of the Affiliated Hospital of Chuanbei Medical College. The studies were conducted in accordance with the local legislation and institutional requirements. The participants provided their written informed consent to participate in this study.

## Author contributions

XL: Investigation, Methodology, Writing – original draft, Writing – review & editing.
